# Functional asymmetry and plasticity of electrical synapses interconnecting neurons through a 36-state model of gap junction channel gating

**DOI:** 10.1371/journal.pcbi.1005464

**Published:** 2017-04-06

**Authors:** Mindaugas Snipas, Lina Rimkute, Tadas Kraujalis, Kestutis Maciunas, Feliksas F. Bukauskas

**Affiliations:** 1 Institute of Cardiology, Lithuanian University of Health Sciences, Kaunas, Lithuania; 2 Department of Mathematical Modeling, Kaunas University of Technology, Kaunas, Lithuania; 3 Department of Applied Informatics, Kaunas University of Technology, Kaunas, Lithuania; 4 Dominick P. Purpura Department of Neuroscience, Albert Einstein College of Medicine, New York City, New York, United States of America; Rush University Medical Center, UNITED STATES

## Abstract

We combined the Hodgkin–Huxley equations and a 36-state model of gap junction channel gating to simulate electrical signal transfer through electrical synapses. Differently from most previous studies, our model can account for dynamic modulation of junctional conductance during the spread of electrical signal between coupled neurons. The model of electrical synapse is based on electrical properties of the gap junction channel encompassing two *fast* and two *slow* gates triggered by the transjunctional voltage. We quantified the influence of a difference in input resistances of electrically coupled neurons and instantaneous conductance–voltage rectification of gap junctions on an asymmetry of cell-to-cell signaling. We demonstrated that such asymmetry strongly depends on junctional conductance and can lead to the unidirectional transfer of action potentials. The simulation results also revealed that voltage spikes, which develop between neighboring cells during the spread of action potentials, can induce a rapid decay of junctional conductance, thus demonstrating spiking activity-dependent short-term plasticity of electrical synapses. This conclusion was supported by experimental data obtained in HeLa cells transfected with connexin45, which is among connexin isoforms expressed in neurons. Moreover, the model allowed us to replicate the kinetics of junctional conductance under different levels of intracellular concentration of free magnesium ([Mg^2+^]_i_), which was experimentally recorded in cells expressing connexin36, a major neuronal connexin. We demonstrated that such [Mg^2+^]_i_-dependent long-term plasticity of the electrical synapse can be adequately reproduced through the changes of *slow* gate parameters of the 36-state model. This suggests that some types of chemical modulation of gap junctions can be executed through the underlying mechanisms of voltage gating. Overall, the developed model accounts for direction-dependent asymmetry, as well as for short- and long-term plasticity of electrical synapses. Our modeling results demonstrate that such complex behavior of the electrical synapse is important in shaping the response of coupled neurons.

## Author summary

In most computational models of neuronal networks, it is assumed that electrical synapses have a constant and ohmic conductance. However, numerous experimental studies demonstrate that connexin-based channels expressed in neuronal gap junctions can change their conductance in response to a transjunctional voltage or various chemical reagents. In addition, electrical synapses may exhibit direction-dependent asymmetry of signal transfer. To account for all these phenomena, we combined a 36-state model of gap junction channel gating with Hodgkin–Huxley equations, which describes neuronal excitability. The combined model (HH-36SM) allowed us to evaluate the kinetics of junctional conductance during the spread of electrical signal or in response to chemical factors. Our modeling results, which were based on experimental data, demonstrated that electrical synapses exhibit a complex behavior that can strongly affect the response of coupled neurons. We suggest that the proposed modeling approach is also applicable to describe the behavior of cardiac or other excitable cell networks interconnected through gap junction channels.

## Introduction

In most models of neuronal networks, it is assumed that electrical synapses exhibit constant conductance, and that electric synaptic transmission is bidirectional and symmetric. However, experimental studies show that these assumptions are not always satisfied. For instance, some synapses formed of gap junction channels exhibit an instantaneous conductance–voltage rectification, which promotes a direction-dependent asymmetry of electrical signaling [1–4]. In addition, all members of the connexin (Cx) family forming gap junction channels exhibit a sensitivity of junctional conductance to the transjunctional voltage [5]. Moreover, voltage sensitivity of gap junctions can be strongly affected by chemical factors, e.g. by intracellular concentrations of H^+^, Ca^2+^ or Mg^2+^ [6–8]. Thus, electrical synapses are not just passive pores, but can exhibit dynamic changes of junctional conductance. Presumably, these changes in electrical synaptic strength could affect the transfer of an electrical signal. The purpose of our study was to develop a computational model for evaluation of such an interaction between electrical synapses and signal transmission between coupled neurons.

The first quantitative models describing equilibrium [9, 10] and kinetic [11] properties of junctional conductance dependence on transjunctional voltage were based on the assumption that the channel can be in two states, open and closed. Later, single channel studies have shown that transjunctional voltage causes channels to close to a subconductance (residual) state [12, 13] with *fast* gating transitions, and to a fully closed state with *slow* gating transitions [14, 15]. Thereafter, it was proposed that gap junction channels comprise two types of gating mechanisms, *fast* and *slow*, each exhibiting rectification of their unitary conductances depending on the voltage across them. These properties were described in a stochastic 16-state model (16SM) of gap junction channel gating [16] in which *fast* and *slow* gates operate between open (o) and closed (c) states. However, experimental data from our and other groups [17, 18] allowed us to suggest that the *slow* gate operates between open (o), initial-closed (c_1_) and deep-closed (c_2_) states. Such a suggestion was implemented in a 36-state model (36SM) of voltage gating [19]. The 36SM allowed us to reproduce experimentally observed gating behavior of gap junction channels more adequately than 16SM, especially regarding the kinetics of conductance recovery, or a low fraction of functional channels clustered in junctional plaques.

Earlier [20], we combined a 16SM of gap junction channel gating and rectification with the Hodgkin–Huxley (HH) equations [21]. The developed model (HH-16SM) allowed us to evaluate the kinetics of junctional conductance during the spread of excitation in neuronal networks. In this study, we replaced 16SM with 36SM for a better evaluation of junctional conductance kinetics. We applied the combined model (HH-36SM) to investigate the signal transfer between electrically coupled neurons in response to different types of presynaptic inputs, such as electrotonic signals or action potentials (APs).

In this study, we analyse three main aspects of the 36SM with respect to the functional behavior of electrical synapses. Firstly, transjunctional voltage distribution across each channel gate can result to almost instantaneous asymmetric conductance-voltage rectification of gap junction channel. We showed that such rectification of gap junctions can affect the asymmetry of the electrical cell-to-cell signaling, especially the spread of a single AP.

Secondly, in the 36SM, the gap junction channel can transit between open and closed states, and probabilities of these transitions depend on voltage across each channel gate. We demonstrated that closing of gap junction channels could be induced by transjunctional voltage spikes, which develop during the spread of excitation. More precisely, our modeling results showed that voltage spikes induced by the trains of APs can cause an accumulation of gap junction conductance decay. As a result, the junctional conductance can significantly decrease in just a few seconds, and substantially modulate electrical signaling between neurons. This short-term plasticity of electrical synapses was supported by our electrophysiological experiments in HeLa cells expressing connexin45.

Thirdly, we suggested that some types of chemical modulation of electrical synapses could be explained by an assumption that the values of 36SM parameters depend on chemical factors. Under such a hypothesis, the chemical modulator would influence the junctional conductance by modifying voltage sensitivity properties of a gap junction channel. In this case, the chemically-induced variation of junctional conductance would be explained by the changed equilibrium of open and closed voltage sensitive channels, and not by a separate chemical gate. To illustrate the feasibility of this idea, we fitted the 36SM to explain the kinetics of connexin36 gap junctional conductance under different concentrations of free magnesium ions ([Mg^2+^]_i_). We demonstrated that a long-term (a few minutes) plasticity, which is induced by variation in [Mg^2+^]_i_, can be adequately reproduced through the changes of 36SM parameters.

Thus, the presented model accounts for the complex behavior of electrical synapses under a wide variety of voltage and temporal conditions. Moreover, all these phenomena can be explained by the underlying mechanisms of gap junction channel voltage gating. Such a modeling approach allows one to evaluate the response of neuronal networks, which would be very difficult to measure experimentally.

## Materials and methods

### 36 state model (36SM) of gap junction channel voltage gating

**Fig 1:**
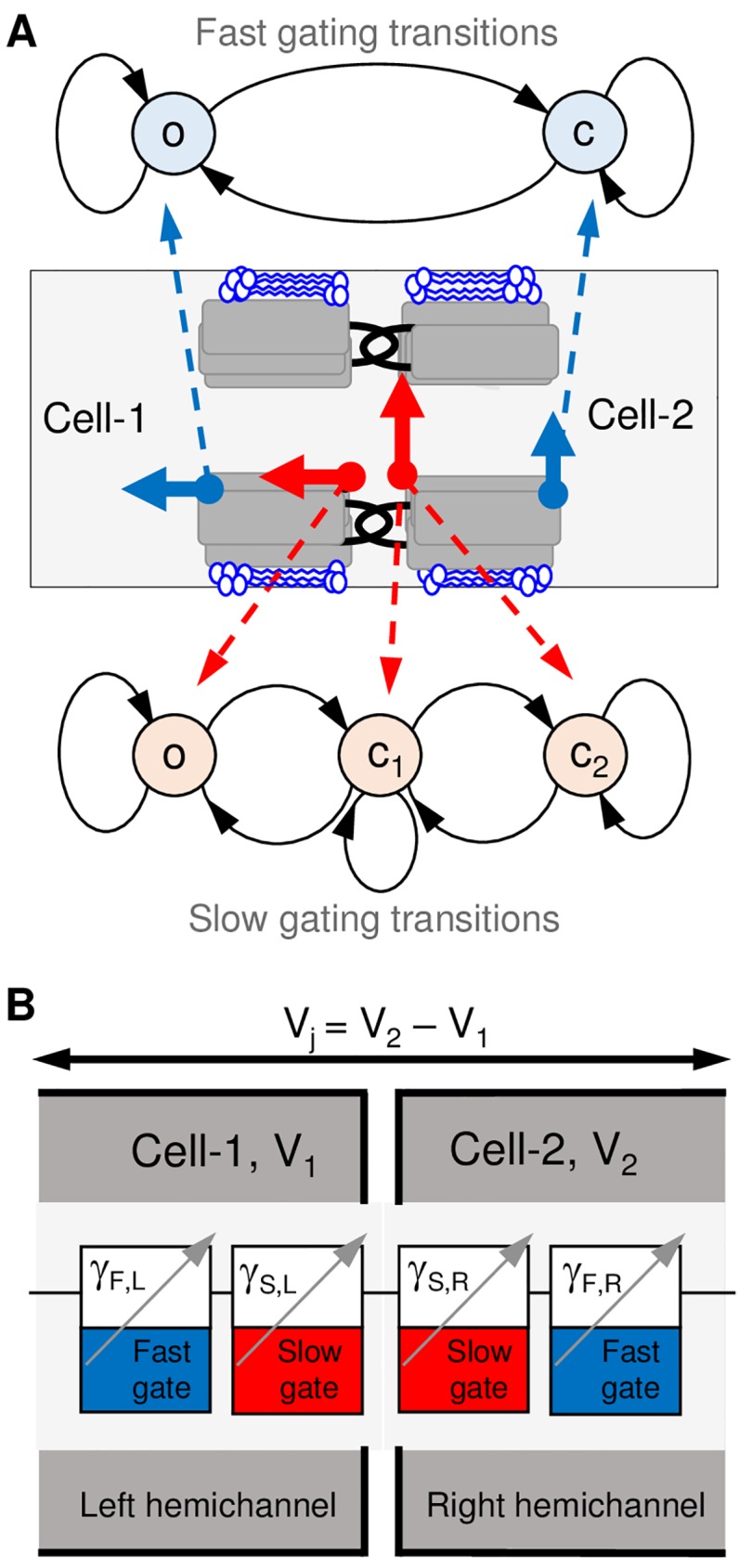
A schematic of 36SM. A) Schematic of gap junction channel enclosing *fast* (blue arrows) and *slow* (red arrows) gates and their gating transitions. The *fast* gate can be in the open (o) or closed (c) states. The closed state of the *fast* gate exhibits residual conductance. The slow gate can reside in open (o) or in one of two fully closed states: initial-closed (c_1_) or deep-closed (c_2_). Gating probabilities depend on the transjunctional voltage (V_j_), which is evaluated as a difference between membrane voltages of two adjacent cells (V_1_ and V_2_). B) Schematic of four gates arranged in series in a gap junction channel. Voltage distribution across each gate depends on their unitary conductances, which can rectify.

Junctional conductance of electrical synapses was evaluated using a Markov chain 36-state model of voltage gating, which is detailed in [19]. The model describes the probabilistic behavior of gap junction channels in response to the transjunctional voltage. In the 36SM, the gap junction channel consists of two hemichannels, each enclosing one *fast* and one *slow* gates (Fig 1). Thus, the channel comprises four gates (*fast left*, *slow left*, *slow right* and *fast right*), all arranged in series (Fig 1B). The *fast* and the *slow* gates operate according to a linear kinetic schemes, *o*↔*c* and *o*↔*c*_1_↔*c*_2_, respectively (Fig 1A). Thus, gap junction channel can be in 36 (2∙3∙3∙2) different states, and overall junctional conductance is estimated as an averaged value of each state conductance weighted to their probabilities. Transition probabilities between system states depend on transjunctional voltage distribution across gates, which must be evaluated first. In general, the voltage distribution can be nonlinear due to rectification of unitary conductances of channel gates.

#### Evaluation of conductance–voltage rectification of gap junction channel using 36SM

Since all channel gates are arranged in series, the transjunctional voltage, V_j_, is the sum of voltages across each gate:

$$V_{j}=V_{F,L}+V_{S,L}+V_{S,R}+V_{F,R}.$$ (1)

The conductance of a single gap junction channel, *γ*_*j*_, at a given state is evaluated as follows:

$$\gamma_{j}=\frac{1}{\left(\frac{1}{\gamma_{\text{F},\text{L}}}+\frac{1}{\gamma_{\text{S},\text{L}}}+\frac{1}{\gamma_{\text{S},\text{R}}}+\frac{1}{\gamma_{\text{F},\text{R}}}\right)}.$$ (2)

Here *γ*_*F*,*L*_, *γ*_*S*,*L*_, *γ*_*S*,*R*_ and *γ*_*F*,*R*_ are unitary conductances of channel gates at a given state. For the non-rectifying channel, voltages across each gate can be estimated in a single step as follows:

$$V_{F,L}=\frac{V_{j}\cdot \gamma_{F,L}}{\gamma_{j}},\;V_{S,L}=\frac{V_{j}\cdot \gamma_{S,L}}{\gamma_{j}},\;V_{S,R}=\frac{V_{j}\cdot \gamma_{S,R}}{\gamma_{j}}\;\mathrm{and}\;V_{F,R}=\frac{V_{j}\cdot \gamma_{F,R}}{\gamma_{j}}.$$ (3)

Typically, all connexins exhibit greater or lesser degrees of instantaneous conductance-voltage rectification. It was proposed that such rectification results from positively or negatively charged amino acids lining the channel pore [17]. Because this process is based on electric field properties, for our modeling purposes, it can be assumed to act instantaneously. To reproduce such instantaneous rectification of gap junction channel, we supposed that unitary conductances of gates rectify according to an exponential function of voltage as in [22]. For example, conductance of the *fast* left gate, *γ*_*F*,*L*_, is defined as

$$\gamma_{F,L}=\gamma_{F,L;0}\cdot e^{V_{F,L}/R_{F,L}};$$ (4)

here *γ*_*F*,*L*;0_ is *γ*_*F*,*L*_ at *V*_*F*,*L*_ = 0. Rectification of gates does not allow for the evaluation of V_F,L_, V_S,L_, V_S,R_ and V_F,R_ in a single step as in (3). For that, (1)–(4) are iterated until the *γ*_*j*_ value converges. Then, overall instantaneous junctional conductance, g_j_, is estimated as a weighted average of all 36 state conductances. A more detailed description is presented in [19].

The emerging conductance–voltage (g_j_–V_j_) relationship of a gap junction is non-exponential. For example, if gates of the left and right hemichannels have equal rectification coefficients of opposite polarities (R_F,L_ = R_S,L_ = R_L_ = –R_R_ = –R_S,R_ = –R_F,R_), then the g_j_–V_j_ curve is symmetric and has the following shape:

$$g_{j}=\frac{1}{C_{1}e^{-C_{2}\frac{V_{j}}{R_{L}}}+C_{3}e^{C_{4}\frac{V_{j}}{R_{R}}}}.$$ (5)

**Fig 2:**
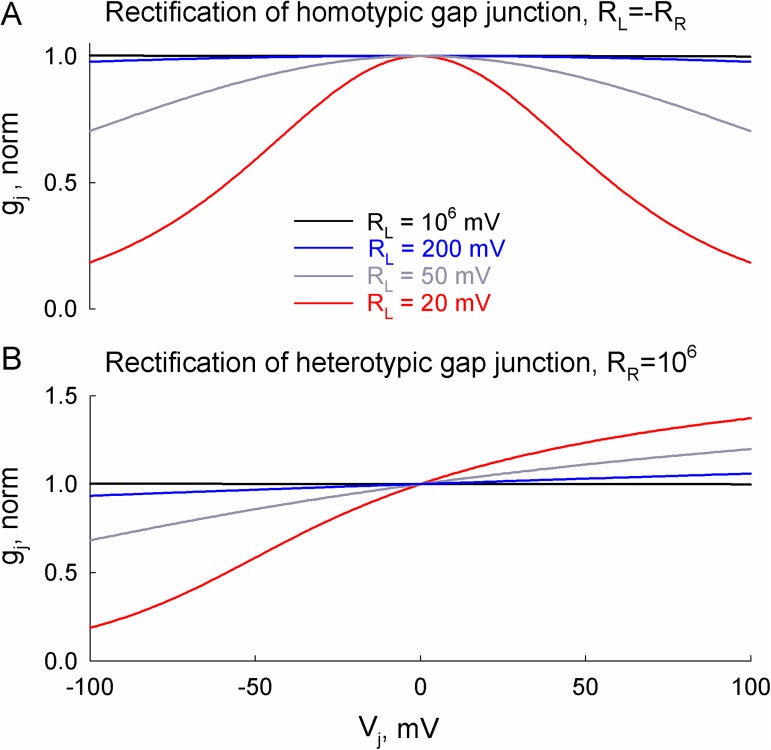
Conductance–voltage (g_j_–V_j_) rectification in the 36SM. (A) Emerging rectification curves of homotypic gap junction; in this case, rectification coefficients on the left and right hemichannels were equal but of opposite signs, R_L_ = –R_R_. (B) The g_j_–V_j_ relationships of heterotypic gap junction; g_j_ asymmetry at opposite V_j_ polarities was obtained by setting R_R_ to a very large value (10^6^ mV), while R_L_ was varied as in (A). The g_j_ values were normalized at V_j_ = 0 mV.

Here, the constants C_1_, C_2_, C_3_ and C_4_ are evaluated during iterative estimation of g_j_. Fig 2 shows normalized junctional conductance of homotypic (A) and heterotypic (B) gap junctions. Examples of symmetric g_j_–V_j_ relationships at different values of rectification coefficient R_L_ are presented in Fig 2A.

In the 36SM, an asymmetric conductance–voltage relationship can be obtained by setting different values of R_L_ and R_R_. For example, at large values of R_R_ the emerging g_j_ resembles a logistic function:

$$g_{j}=\frac{1}{1+C_{1}e^{-C_{2}\frac{V_{j}}{R_{L}}}}.$$ (6)

Examples of g_j_s at different values of rectification coefficient R_L_ are presented in Fig 2B. For example, at R_L_ = 20 mV conductance–voltage rectification of the gap junction channel is comparable to that observed in heterotypic Cx32/Cx26 junctions [17].

#### Probabilities of a voltage gating transitions

Once voltage distribution across each gate is evaluated, transition probabilities of channel gates can be estimated. In the 36SM, probabilities of *fast* gate transitions are given by

$$p_{o\to c}=\frac{P_{t}\cdot K}{\left(1+K\right)},\;p_{o\to o}=1-p_{o\to c},\;p_{c\to o}=\frac{P_{t}}{\left(1+K\right)}\;\mathrm{and}\;p_{c\to c}=p_{c\to o},$$ (7)

where *K* is the equilibrium constant of *slow* or *fast* gates [23] and *P*_*t*_ adjusts simulated and experimental times [16, 24]. For example, for a *fast* gate, *K* is defined as follows

$$K_{F}=e^{A_{F}\left(\Pi \cdot V_{F}-V_{F,0}\right)};$$ (8)

here *A*_*F*_ characterizes sensitivity to the voltage of the *fast* gate, V_*F*_ is the voltage across the *fast* gate, V_*F*,0_ is the voltage across the *fast* gate at which *K*_*F*_ is equal to 1, and Π is its gating polarity (+1 or –1). Parameters *A*_*F*_,V_*F*,0_ and Π depend on connexin type.

The *slow* gate *o*↔*c*_1_↔*c*_2_ transitions are given by

$$p_{o\to c1}=\frac{P_{t}\cdot K}{\left(1+K\right)},\;p_{o\to o}=1-p_{o\to c1},\;p_{c1\to o}=\frac{P_{t}\left(1-p_{c1\to c2}\right)}{\left(1+K\right)},$$

$$p_{c1\to c1}=1-p_{c1\to o}-p_{c1\to c2},\;p_{c1\to c2},\;p_{c2\to c1}\;\mathrm{and}\;p_{c2\to c2}=1-p_{c2\to c1};$$ (9)

here *p*_*c*1→*c*2_ and *p*_*c*2→*c*1_ do not depend on voltage but on connexin type and on cytosolic or surrounding conditions [19].

Transition probabilities between all 36 states of the channel are estimated as a product of transition probabilities of individual gates. All these probabilities combine into a transition probability matrix **P** consisting of 36 × 36 entries. The vector **p**^(*n* + 1)^, containing probabilities of states at a discrete time step (*n* + 1), can be estimated from recursive relation

$$p^{\left(n+1\right)}=p^{\left(n\right)}\cdot P.$$ (10)

Then, the junctional conductance of a gap junction channel is estimated as an expected value of all 36 state conductances. A more detailed description is presented in [19].

Voltage gating of gap junctions results to a steady-state conductance–voltage relationship [16], which is often described by the Boltzmann equation [9]. Presumably, such process of channel gating requires conformational changes of connexins, and it usually takes a few or more seconds to reach a steady state. Therefore, in electrophysiological studies, a clear distinction should be made between conductance–voltage rectification that is virtually *instantaneous*, and the *steady-state* relationship, which results from voltage gating of gap junction channels (see Fig 1 in [17] or Fig 6 in [25]). In this study, we reserve the term *rectification* only for an *instantaneous* conductance–voltage relationship.

### Neuronal excitability

#### Hodgkin-Huxley model

Membrane excitability properties were described using Hodgkin–Huxley equations as per the original publication [21], but with resting potential shifted to –70 mV. In describing excitability of neurons, we accounted for the presence of junctional current in parallel with the ionic channel and leak currents. A detailed description is presented in S1 Text. We used Euler’s method for numerical solution of the ordinary differential equations, which was implemented in MATLAB.

#### Passive membrane properties

An input resistance of neurons was evaluated from changes in the transmembrane potential in response to an applied external current in a single neuron. To change the input resistance, we varied the surface area of the cell in Hodgkin–Huxley model. This resulted in proportional changes in membrane capacitance. For a more detailed description, we refer to S1 Text.

### HH-36SM

The developed HH-36SM combines Hodgkin–Huxley equations that describe excitability of neurons and a 36-state model (36SM) of gap junction channel gating that evaluates conductance of the electrical synapse. More precisely, membrane voltages of the neurons are estimated using the Hodgkin-Huxley model. The resulting transjunctional voltage can affect the junctional conductance, which is evaluated using the 36SM. Thus, the HH-36SM allowed us to simulate electrical signal transfer between neurons connected through modulatable gap junctions.

### Electrophysiological experiments

#### Cell lines and culture conditions

Experiments were performed in HeLa cells (human cervical carcinoma, ATCC CCL-2), transfected with Cx45, and RIN cells (rat β-cell insulinoma, ATCC CRL-2057) transfected with Cx36. Cx36 was tagged with enhanced green fluorescent protein, EGFP, which allowed us to select cell pairs expressing junctional plaques representing clusters of gap junction channels [26]. HeLa and RIN cell cultures were grown in DMEM and RPMI 1640 media, respectively, supplemented with 8% fetal calf serum, 100 mg per ml streptomycin and 100 units per ml penicillin, and maintained in a CO_2_ incubator (37°C and 5% CO_2_).

#### Electrophysiological measurements

Electrophysiological recordings were performed in a modified Krebs–Ringer solution containing the following (in mM): 140 NaCl, 4 KCl, 2 CaCl_2_, 1 MgCl_2_, 2 CsCl, 1 BaCl_2_, 5 glucose, 2 pyruvate, 5 HEPES, pH 7.4. Recording pipettes (3–5 MΩ) were filled with standard pipette solution containing the following (in mM): 130 CsCl, 10 NaAsp, 1 MgCl_2_, 0.26 CaCl_2_, 2 EGTA and 5 HEPES (pH 7.2). To change the intracellular free magnesium concentration from 1 to 0.01 or 5 mM, we used pipette solutions containing different concentrations of MgCl_2_ calibrated according to Maxchelator software.

Junctional conductance was measured in selected cell pairs using a dual whole-cell patch-clamp system [27]. Each cell within a pair was voltage clamped with a separate patch-clamp amplifier (EPC-7plus, HEKA). V_j_ was induced by stepping the voltage in one cell while keeping it constant in the other. Junctional current (I_j_) was measured as the change in the current of a neighboring cell and g_j_ was estimated from the relationship g_j_ = I_j_/V_j_. To measure g_j_ changes, we applied repeated (1 Hz) bipolar V_j_ ramps of 400 ms in duration and of low amplitude (±10 mV) to avoid a direct V_j_ effect on gating.

Signals were acquired and analyzed using an analog-to-digital converter (National Instruments, Austin, TX) and custom-made software [28].

## Results

### Rectification of gap junction channels and asymmetry in electrical synaptic transmission

#### Asymmetry in electrical synaptic transmission depending on input resistances of coupled neurons

**Fig 3:**
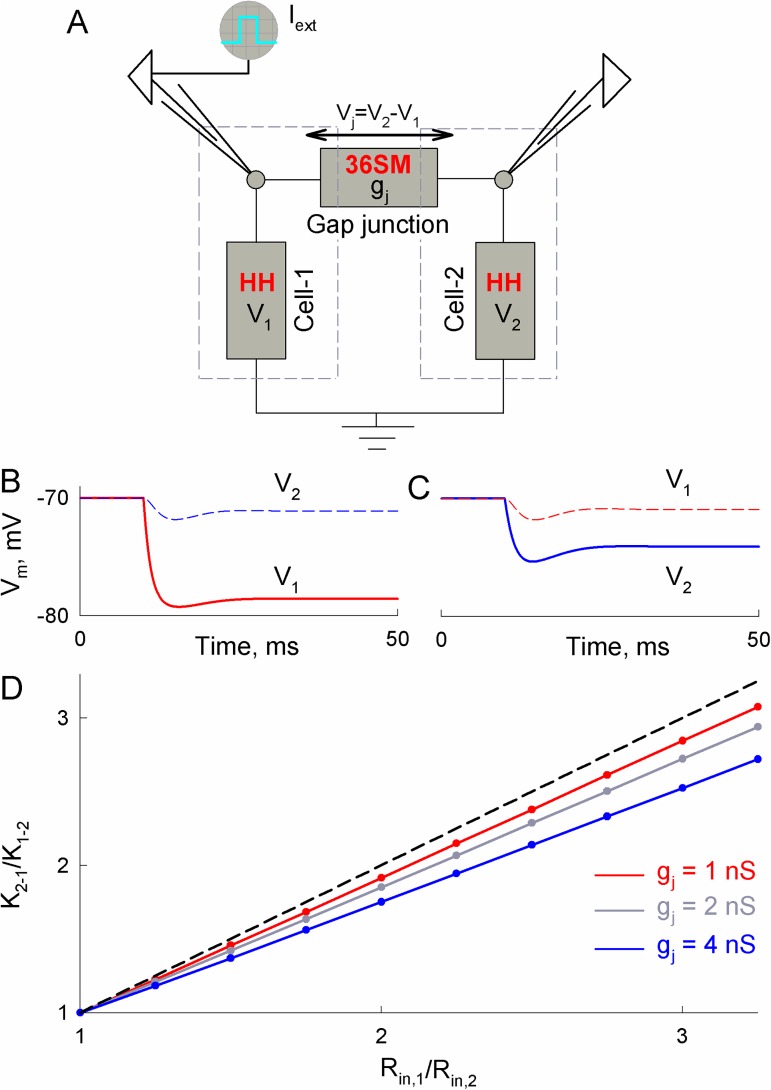
Asymmetry of electrical coupling between two neurons with different input resistances, R_in,1_ and R_in,2_. A) An electrical scheme of two cells connected through soma-somatic gap junctions. Membrane voltages of cell-1 and cell-2 (V_1_ and V_2_) are described by Hodgkin–Huxley equations (HH). Junctional conductance (g_j_) depends on transjunctional voltage (V_j_) and is estimated from the 36-state model (36SM) of gap junction channel gating. An external current (I_ext_) can be applied to either of the cells. B–C) Simulated changes of transmembrane potential (V_m_) in cell-1 and cell-2 during the external current step of –100 pA applied to cell-1 (B) or cell-2 (C); here R_in,1_/R_in,2_ = 2 and the junctional conductance (g_j_) was equal to 4 nS. D) The dependence of coupling asymmetry, K_2-1_/K_1-2_, on the ratio of input resistances, R_in,1_/R_in,2_, at different g_j_s; here K_1-2_ = V_2_/V_1_ and K_2-1_ = V_1_/V_2_.

Studies of gap junctional communication in brain slices are typically performed by injecting a hyperpolarizing current step into one cell and measuring the response in a neighboring cell. Coupling coefficients are estimated as the ratio of electrotonic responses in neighboring and stimulated cells, K_1-2_ = V_2_/V_1_ or K_2-1_ = V_1_/V_2_ [29]. If K_1-2_ and K_2-1_ are not equal, then this shows an asymmetry of gap junctional communication, which can arise due to rectification of gap junction channels or the difference in input resistances (R_in_). We simulated electrotonic signal transfer between two neurons, connected through the soma-somatic junction using HH-36SM. The schematics of such experiment are presented in Fig 3A. This allowed us to evaluate to what extent coupling asymmetry can be explained by differences in R_in_s. To modulate R_in_, we varied the surface areas of coupled cells (more details in S1 Text). R_in_ of the presynaptic cell was close to 125 MΩ, which is within the range of experimentally measured values in MesV neurons [30]. Gating parameters of a 36SM were chosen to resemble Cx36 [19, 20], a major connexin forming electrical synapses between neurons in the central nervous system.

Fig 3B and 3C show a significant difference between K_1-2_ and K_2-1_ when the input resistance of cell-2 is two times smaller than that of cell-1. The summarized results in Fig 3D show that the ratio K_2-1_/K_1-2_ depends linearly on R_in,1_/R_in,2_, and the slope of the line approaches 1 (dashed line in Fig 3D) when the junctional conductance decreases.

**Fig 4:**
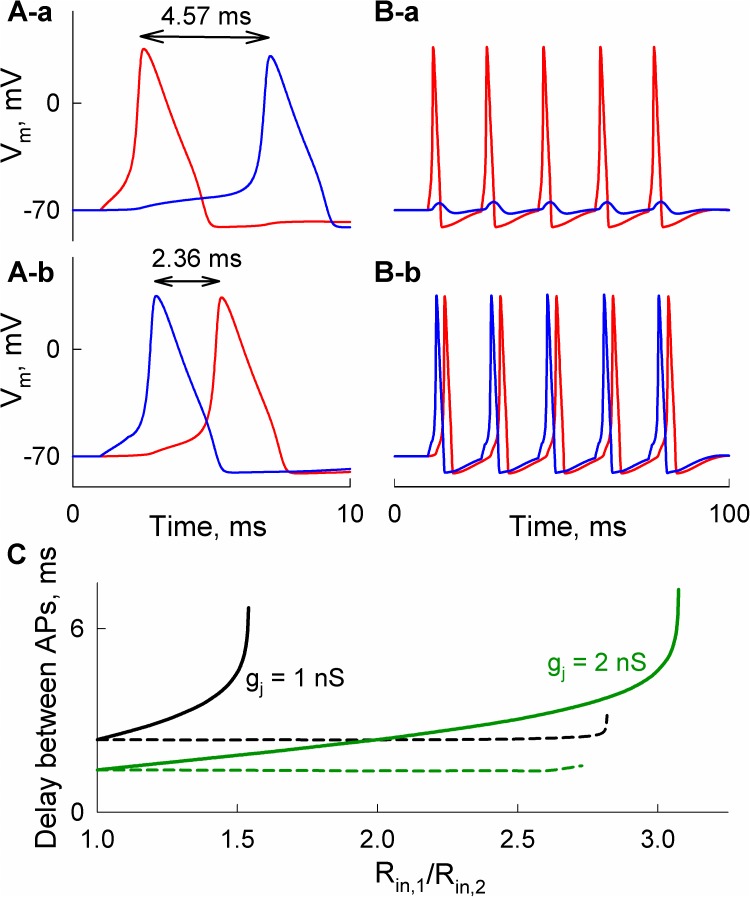
Asymmetry of AP transfer between neurons with different input resistances, R_in,1_ and R_in,2_. A) AP transfer was almost 2-fold longer when a short (1 ms) external stimulus of 250 pA was applied to cell-1 (A-a) than to cell-2 (A-b); R_in,1_/R_in,2_ = 1.5. B) Unidirectional transfer of APs caused by repeated short stimuli between cells with R_in,1_/R_in,2_ = 2. In A and B, junctional conductance g_j_ was equal to 1 nS. C) Delays between APs depending on R_in,1_/R_in,2_ at different g_j_s. Solid and dashed curves were obtained when neurons with higher and lower R_in_ were stimulated, respectively.

To study asymmetry in AP transfer, we applied short depolarizing current pulses and measured the delay between APs in cell-1 and cell-2. Fig 4A shows that a moderate difference in input resistances (R_in,1_/R_in,2_ = 1.5) results in a significant difference in the delay of APs transfer in retrograde (4A-a) and anterograde directions (4A-b). Moreover, at R_in,1_/R_in,2_ = 2, periodic short stimuli applied to cell-1 caused only low amplitude electrotonic responses in cell-2 (Fig 4B-a). In contrast, cell-1 responded with AP to each excitation of cell-2 (Fig 4B-b). Fig 4C shows differences in AP delay anterogradely (solid lines) and retrogradely (dashed lines) depending on R_in,1_/R_in,2_. The right end-points of solid curves correspond to cut-off values of R_in,1_/R_in,2_ under which the spread of APs cannot be transferred from cell-1 to cell-2. Similarly, the end-points of dashed lines show the limit when R_in,1_/R_in,2_ becomes too high for applied stimulus to induce excitation in cell-2.

#### The rectification of gap junction channels and asymmetry in electrical signaling across the gap junction

Another source of asymmetry in electrical synaptic transmission is an instantaneous conductance–voltage rectification of gap junctions. Using the 36SM, asymmetric rectification of junctional conductance can be obtained by setting different values of a rectification coefficient of left and right hemichannels, as described in Materials and Methods. We simulated the transfer of APs between two neurons to evaluate the effect of such asymmetric gap junctional rectification. In this case, both cells had equal input resistances (~125 MΩ).

**Fig 5:**
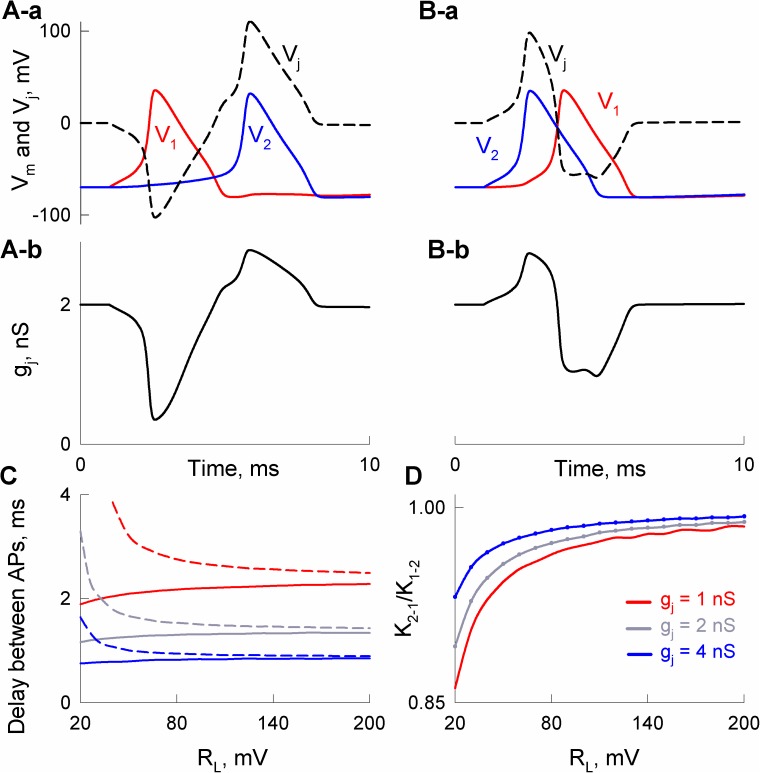
Asymmetry of transjunctional transfer of APs through electrical synapses caused by gap junction channel rectification. A–B) Delays between APs transferred through an electrical synapse composed of rectifying channels (R_L_ = 20 mV). Short external stimuli of 250 pA were applied to cell-1 (A) and cell-2 (B). A-a and B-a show voltages in cell-1 (red) and cell-2 (blue), V_1_ and V_2_, respectively. Dashed lines show transjunctional voltage spikes (V_j_); V_j_ = V_2_ –V_1_. A-b and B-b show resulting changes of junctional conductance (g_j_). C) Dependence of a delay between APs during anterograde (from cell-1 to cell-2; solid curves) and retrograde (dashed curves) excitation transfer on rectification of gap junctions. D) Dependence of electrotonic coupling asymmetry, K_2-1_/K_1-2_, on rectification coefficient, R_L_.

Transjunctional voltage spikes that develop due to a delay between APs in neighboring cells influence conductance of rectifying electrical synapse instantaneously. Moreover, instantaneous changes in junctional conductance can lead to direction-dependent asymmetry in AP transfer, if the gap junction exhibits an asymmetric conductance–voltage rectification as presented in Fig 2B. For example, at R_L_ = 20 mV, the transfer of AP from cell-1 to cell-2 caused an initial decrease in junctional conductance (Fig 5A-b), which resulted in a delay of ~3.3 ms (Fig 5A-a). In contrast, AP spread from cell-2 to cell-1 caused an initial increase in junctional conductance (5B-b), which accelerated AP spread; the delay was equal to ~1.1 ms in this case (Fig 5B-a). Fig 5C shows the dependence of the delay between APs on the rectification coefficient. It can be seen that stronger rectification (smaller R_L_ values) tends to decrease or increase a delay of AP transfer in opposite directions. The effect of such rectification also depends on the values of junctional conductance. For example, the developed decrease of junctional conductance can result in the unidirectional spread of AP at weakly coupled cells.

We also evaluated an effect of gap junction channel rectification on the asymmetry of electrotonic coupling, K_2-1_/K_1-2_ (Fig 5D). Electrotonic signal transfer is less affected by gap junctional rectification than the spread of APs due to much smaller transjunctional voltages which develop during electrotonic coupling measurements. In general, for electrotonic and AP transfer, a functional asymmetry is better expressed among electrical synapses with lower junctional conductances. Our data showed that this applies to asymmetries caused by differences in R_in_s (Figs 3D and 4C) as well as by rectification of gap junction channels (Fig 5C and 5D).

### Voltage gating of gap junction channels and short-term plasticity of electrical synapses

#### Spiking activity-dependent voltage gating and plasticity of electrical synapses

**Fig 6:**
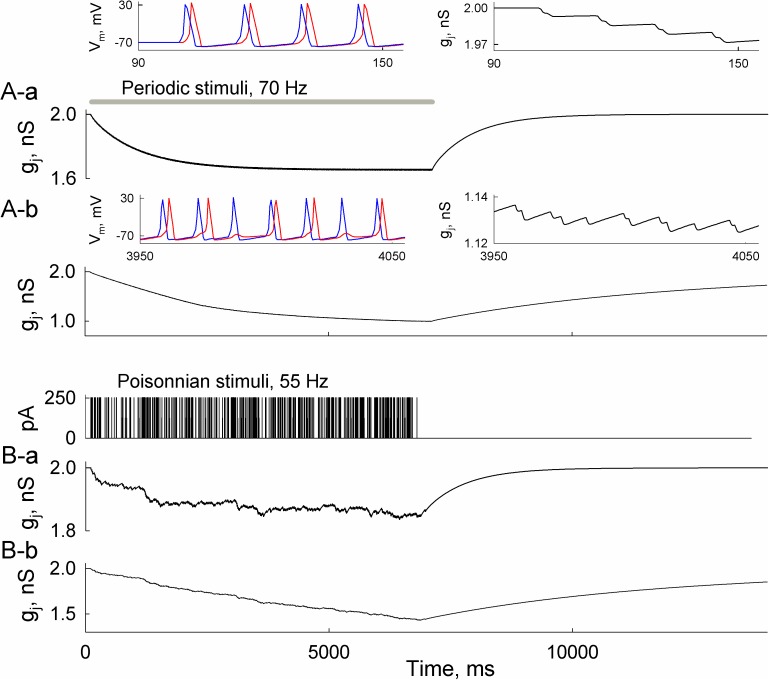
Dynamic changes of junctional conductance depend on voltage sensitivity of gap junctions and neuronal spiking activity. A-a) A decay of junctional conductance, g_j_, during neuronal spiking activity. The grey horizontal line shows the time interval of stimulation using periodic (70 Hz) depolarizing stimuli of 250 pA in amplitude and 1 ms in duration. Left inset shows the developed transmembrane voltages (V_m_) in presynaptic (blue) and postsynaptic (red) cells, while the right inset shows junctional conductance decrease at enhanced resolution. A-b) The same as in A-a, but in a more voltage-sensitive electrical synapse. Voltage sensitivity of the gap junction was enhanced by lowering the deep-closed transition probability p_c2→c1_ from 0.001 to 0.0001. The left inset in A-b shows that transfer of APs alternates due to decreased g_j_. B) The same as in A, but applied stimuli were distributed randomly according to the Poisson law with 55 Hz rate. In B-a, p_c2→c1_ = 0.001, while p_c2→c1_ = 0.0001 in B-b.

We applied the HH-36SM to evaluate the changes of junctional conductance induced by trains of action potentials. We used the same gating parameters of Cx36 as in [20], while deep-closed transition probabilities were taken from [19]. The input resistances of both neurons were equal (~125 MΩ) and gap junctions were non-rectifying (R_L_ = 10^6^, see Fig 2). Short periodic depolarizing stimuli induced trains of APs in a presynaptic cell, which were transmitted to the postsynaptic cell (the left inset in Fig 6A-a). The resulting biphasic voltage spikes caused small decays of junctional conductance (the right inset in Fig 6A-a). Overall, accumulated reduction of synaptic strength reached ~15% in just a few seconds. For a more voltage-sensitive gap junction, which was simulated by changing probabilities of the c_1_↔c_2_ transition, such decrease reached almost 50% (A-b). It caused an alternating AP transfer (see left inset in Fig 6A-b), and slower recovery of junctional conductance.

In Fig 6B, the trains of APs were caused by stimuli distributed according to the Poisson law (6B-a), which more closely resembles neuronal firing rate variability *in vivo* [31]. The rate of the Poisson process was 55 Hz, which is close to the maximum firing rate in the primary motor cortex of a monkey during an arm-reaching task [32]. Overall, Fig 6B shows a similar, although irregular, decrease of synaptic conductance as in Fig 6A. Slightly higher conductances in 6B can be attributed to lower average frequencies of external stimuli. Interestingly, under stimulation of 5 Hz, which is close to background firing rate in the mammalian visual cortex [33], the junctional conductance had enough time to recover between APs and the accumulated decrease was insignificant (see S1 Fig). Thus, the shown data demonstrate that electrical synapses can exhibit short-term (<10 s) plasticity which strongly depends on voltage sensitivity of electrical synapse and spiking activity of neurons.

#### Validation of short-term plasticity by electrophysiological data

In most electrophysiological studies, voltage gating of gap junction channels is examined in response to long (>10 s) voltage steps or ramps. To test whether junctional conductance decrease could be observed in response to very short spikes arising during neural activity, we performed experiments in HeLa cells expressing connexin45 (Cx45). Cx45 forms electrical synapses in both developing and adult mammalian brain [34], and is also abundantly expressed in the conduction system of the heart [35].

**Fig 7:**
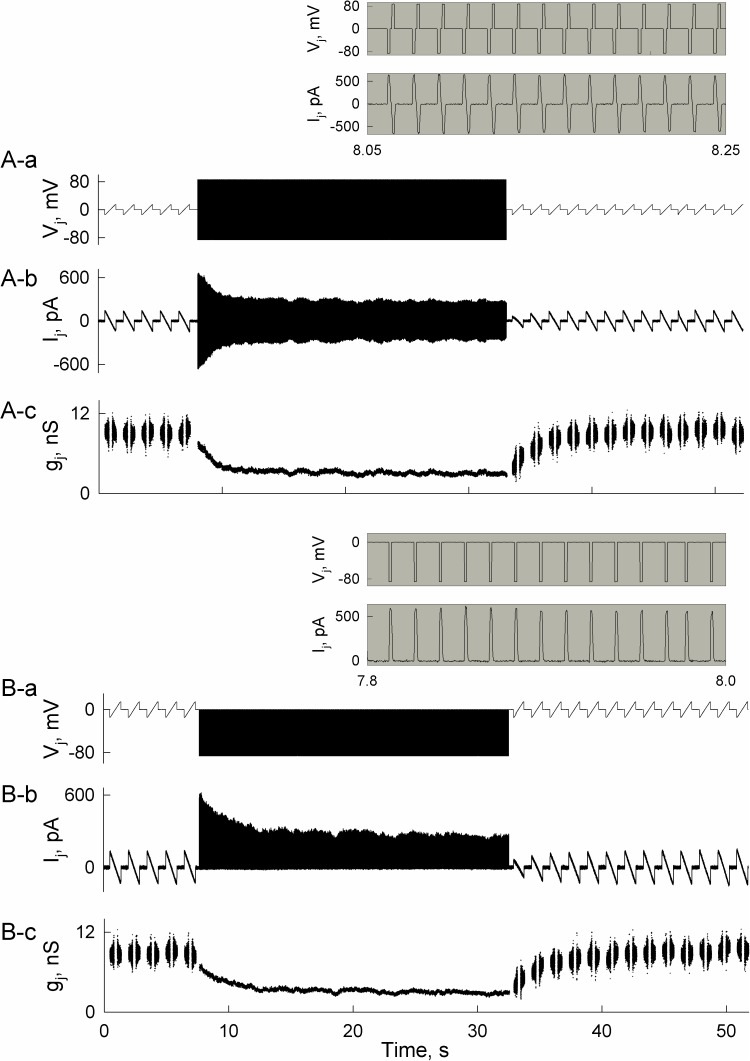
Changes of junctional conductance in HeLa cells expressing Cx45 during bipolar and monopolar stimulation. A) Junctional current (I_j_) and conductance (g_j_) records obtained in response to repeated bipolar voltage (V_j_) stimuli of –85 and +85 mV in amplitude; amplitude and duration of voltage spikes and current responses can be seen in the insets. B) The same as in (A) but unipolar –85 mV instead of bipolar stimuli were applied. Insets (grey background) above 7A and B show applied V_j_ stimuli and registered I_j_ values at enhanced resolution. The values of g_j_ were estimated from g_j_ = I_j_/V_j_.

We generated a series of short periodic voltage steps, which are comparable in their duration and amplitude with transjunctional voltage spikes arising during the spread of APs (see the inset in Fig 7A). We used biphasic and monophasic voltage steps to imitate different types of response in a postsynaptic cell, caused by APs in the presynaptic cell. Biphasic steps resemble voltage spikes observed when APs are transmitted, while monophasic steps develop when the postsynaptic cell responds only with electrotonic depolarization.

Fig 7A-b and 7A-c show a significant decay of junctional current and conductance during repeated bipolar stimuli (7A-a). A decay of junctional conductance during bipolar voltage spikes (see inset in Fig 7A) and its recovery took ~8 s, which is consistent with modeling results shown in Fig 6. Fig 7B-c shows a similar decrease in response to unipolar stimuli applied at the same frequency as in Fig 7A-c.

#### Short-term plasticity of heterotypic gap junctions

**Fig 8:**
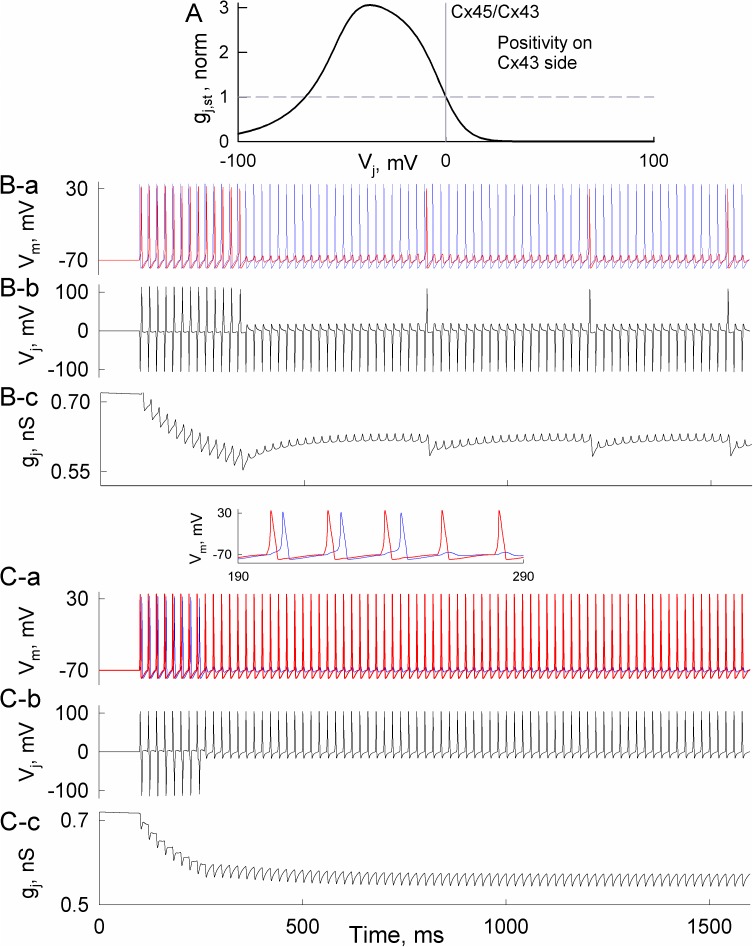
Neuronal activity-dependent changes of junctional conductance in heterotypic gap junctions. A) Simulated steady-state conductance–voltage (g_j,st_–V_j_) relationship of heterotypic gap junctions that resembles experimentally measured g_j,st_–V_j_ relationship of Cx43/Cx45 gap junctions. B shows the transfer of action potentials when the cell, virtually expressing Cx45, was stimulated. B-a) Records of membrane voltage responses in cell-Cx45 (blue trace) and cell-Cx43 (red trace). B-b) Transjunctional voltage (V_j_) spikes developed in the electrical synapse. B-c) Changes of junctional conductance (g_j_) caused by the spiking activity of neurons. C-a,b,c) The same as in B-a,b,c, but a cell, virtually expressing Cx43, was stimulated.

Cells expressing different Cx isoforms can form heterotypic gap junctions, which exhibit an asymmetry in voltage gating due to different properties of composing hemichannels [5]. We presumed that such voltage-gating asymmetry could result in direction-dependent electrical signal transfer in the heterotypic synapse. To test this, we chose the parameters of 36SM roughly to resemble Cx43/Cx45, which is among the most examined heterotypic gap junction [36]. The resulting steady-state conductance–voltage relationship exhibits well-expressed asymmetry of gap junction channel gating at opposite voltage polarities (see Fig 8A).

The simulated spread of action potential through heterotypic electrical synapses depended on whether a cell expressing Cx45 or Cx43 was stimulated. Stimulation of cell-Cx45 caused an accumulation of junctional conductance decrease that prevented an active response in cell-Cx43 after ~300 ms (Fig 8B-a). Consequently, developed unipolar voltage spikes with relative negativity on the Cx43 side (8B-b) increased conductance (B-c) and re-invoked AP. Altogether, this resulted in ~20-fold decreased firing rate in cell-Cx43. In contrast, when cell-Cx43 was stimulated (Fig 8C), bipolar voltage spikes reduced junctional conductance that blocked AP transfer at ~240 ms (8C-a). This resulted in unipolar voltage spikes with relative positivity on the Cx43 side that kept AP transfer permanently blocked. Thus, electrical synapse formed of heterotypic gap junctions can exhibit direction-dependent short-term plasticity.

### Chemically modulated gating of gap junction channels and long-term plasticity of the electrical synapse

#### [Mg^2+^]_i_ modulated gating of electrical synapses

Although it is well established that gap junctional communication can be regulated by various chemical factors, to our knowledge, there is no established mechanistic model to describe chemical or chemically modulated gating. Experimental data show that an effect of chemical uncouplers can be enhanced or reduced by voltage [37–39]. In addition, voltage sensitivity of electrical synapses is affected by chemical factors, such as calcium [7] or magnesium [8]. This suggests that some chemical factors could act through the same underlying mechanisms as voltage gating. We implemented such an idea to reproduce the [Mg^2+^]_i_ effect on connexin36 (Cx36) gap junctions using 36SM.

**Fig 9:**
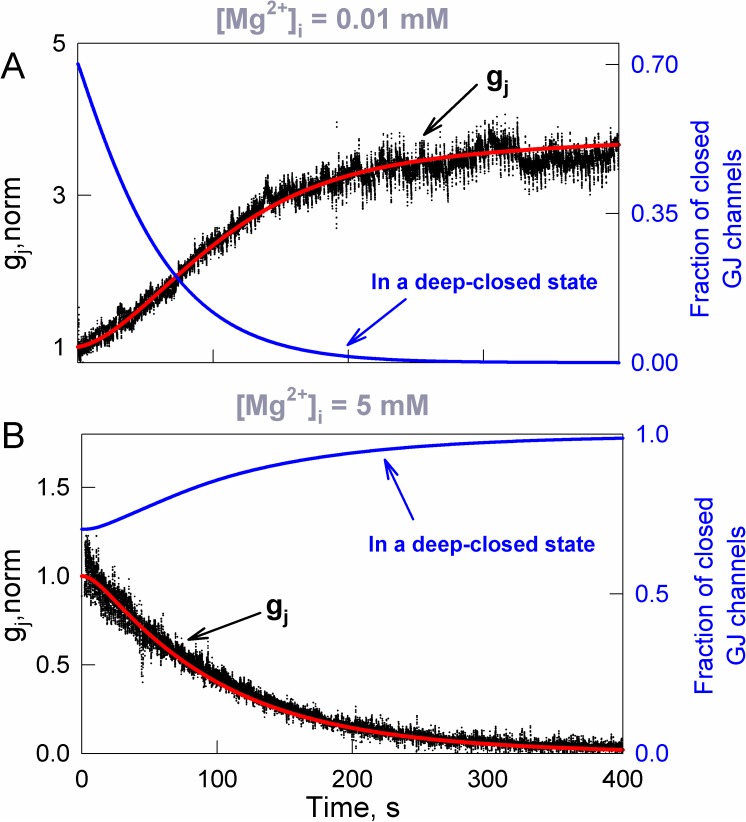
Simulation of [Mg^2+^]_i_–mediated changes of junctional conductance (black traces) measured in RIN cells expressing Cx36. A) [Mg^2+^]_i_ decreased from 1 to 0.01 mM. The fitted junctional conductance (g_j_) kinetics (red trace) was obtained at ~4.5-fold V_0_ increase and ~1000-fold p_c1→c2_ decrease. Blue traces and the right Y axis show the fraction of closed gap junction channels in which at least one *slow* gate is in a deep-closed state (solid blue trace). B) The same as in A, but [Mg^2+^]_i_ increased from 1 to 5 mM. The fitted red trace was obtained when V_0_ decreased ~2 fold, while p_c1→c2_ rose from 0.7 to 0.95. In both A and B, g_j_ values were normalized at [Mg^2+^]_i_ = 1 mM.

Studies in cells and brain slices reported that low [Mg^2+^]_i_ increases, while high [Mg^2+^]_i_ decreases conductance of Cx36 [8]. Fig 9 shows an example of simulated junctional conductance kinetics (red curve) fitted to experimental data (black trace). Model fitting was performed, assuming that variation in [Mg^2+^]_i_ changes the values of the voltage-gating parameters in the 36SM. In Fig 9A, a rise in junctional conductance under [Mg^2+^]_i_ decrease from 1 to 0.01 mM was obtained from a slow increase in V_0_ and sudden drop in the ratio of probabilities, p_c1→c2_/p_c2→c1_; more details are presented in S2 Text. Under [Mg^2+^]_i_ increase from 1 to 5 mM (Fig 9B), the fitting showed opposite tendencies–a decrease in V_0_ and a rise in p_c1→c2_/p_c2→c1_. A more detailed analysis of modeling results revealed that the kinetics of junctional conductance were mainly defined by gating transitions between initial and deep-closed states of the *slow* gate. For example, under [Mg^2+^]_i_ increase, the fraction of gap junction channels in which at least one *slow* gate resides in a deep-closed state decreased from initially ~0.7 to less than 0.01 (right Y axis and solid blue curve in 9A). In contrast, under [Mg^2+^]_i_ decrease, this fraction approached 0.99 (solid blue curve in 9B). These results support the hypothesis that chemical gating could be executed through the *slow* voltage sensitive gate.

**Fig 10:**
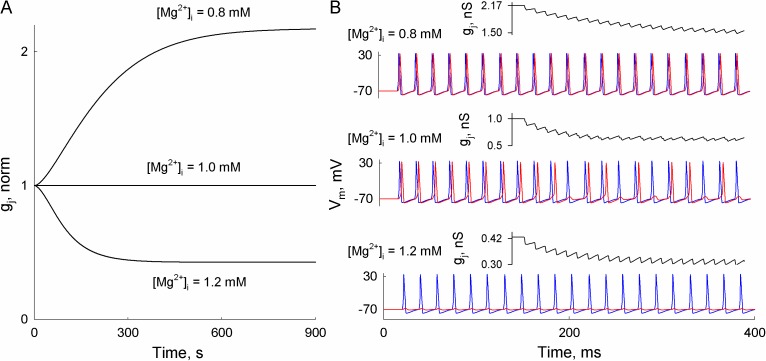
The simulated effect of intracellular free magnesium ion concentration ([Mg^2+^]_i_) on junctional conductance and the spread of excitation through electrical synapse formed of Cx36. A) Simulated kinetics of junctional conductance (g_j_) of electrical synapse formed of Cx36 under different levels of [Mg^2+^]_i_. The parameters of 36SM at [Mg^2+^]_i_ = 0.8 and 1.2 mM were approximated from optimized values, as presented in S2 Text and S2 Fig. The values of g_j_ were normalised at [Mg^2+^]_i_ = 1.0 mM. B) The resulting responses between two coupled neurons at steady-state g_j_ reached at [Mg^2+^]_i_ = 0.8, 1.0 and 1.2 mM. At [Mg^2+^]_i_ = 0.8 mM, ~2.2-fold increased junctional conductance (~2.2 nS) was sufficient to invoke a postsynaptic response (red curves) to each AP in the presynaptic cell (blue curves). The spread of APs was not disturbed by voltage-gating induced g_j_ decrease (see the upper inset in 10B). At [Mg^2+^]_i_ = 1.0 mM, the steady-state g_j_ value of ~1 nS was sufficient to transfer each AP initially, but the decreased g_j_ caused a 2-fold lower firing rate in the postsynaptic cell, as compared with [Mg^2+^]_i_ = 0.8 mM. At [Mg^2+^]_i_ = 1.2 mM, junctional conductance is decreased ~2.5 fold. Such synaptic strength could only invoke an electrotonic response in the postsynaptic cell.

To evaluate the effect of [Mg^2+^]_i_ variation on the spread of APs, we performed a simulation using the HH-36SM. First, we evaluated the conductance kinetics of Cx36 gap junction at more physiological ranges of [Mg^2+^]_i_ (between 0.8 and 1.2 mM [40]). Parameters of the 36SM at [Mg^2+^]_i_ = 0.8 and [Mg^2+^]_i_ = 1.2 mM were roughly approximated from global optimization results at [Mg^2+^]_i_ = 0.01 and 5 mM, as detailed in S2 Text (see also S2 Fig). Modeling results showed (Fig 10A) that junctional conductance increases ~2.2 times under [Mg^2+^]_i_ = 0.8 mM, and decreases ~2.5-fold under [Mg^2+^]_i_ = 1.2 mM, as compared with an initial synaptic strength under [Mg^2+^]_i_ = 1 mM. The kinetics of junctional conductance variation under [Mg^2+^]_i_ is much longer than those induced by spiking activity, and a new steady state level of junctional conductance is maintained for as long as respective [Mg^2+^]_i_ is present. [Mg^2+^]_i_-induced changes in junctional conductance can strongly affect the electrical signal transfer. For example, our results showed a significant facilitation of AP transfer at 0.8 and a strong inhibition at 1.2 mM of [Mg^2+^]_i_ (see Fig 10B). Overall, we suggest that the 36SM can account for some types of chemical modulation of gap junctions, and that such a modeling approach could be applied in simulation of long term plasticity of electrical synapses.

## Discussion

### Functional asymmetry of electrical synaptic transmission and its role in shaping the behavior of a neuronal network

Asymmetry of electrical synaptic transmission has been observed in numerous studies [41–44]. Such asymmetry might arise due to differences in input resistances (R_in_s) of coupled neurons [29], even when gap junctions themselves are symmetric. R_in_ depends on the conductivity of the plasma membrane and its surface area, as well as on the number of neighboring neurons connected through electrical synapses. Another source of electrical synaptic transmission asymmetry is related to instantaneous conductance–voltage rectification of gap junction channel, which results from the inhomogeneous distribution of charged amino acids lining the pore [45]. Such rectification of gap junction channels typically arises in heterotypic junctions under normal conditions [4, 17, 46], but it can also develop in homotypic gap junctions under an asymmetry of intracellular milieu, e.g. gradients of [Mg^2+^]_i_ [47]. In addition, electrical signaling asymmetry across heterotypic channels can arise with repeated stimulation due to voltage gating (see Fig 7). This type of asymmetry in electric synaptic transmission is not instantaneous and depends on past history. The other factors that contribute to asymmetry of signaling do not have this property.

Our data show that asymmetry in electrotonic cell-to-cell communication is more affected by the difference in R_in_s of coupled cells (see Figs 3D and 5D), while gap junctional rectification primarily influences an asymmetry of AP transfer between neurons (Fig 5A–5C). This can be explained by the conductance–voltage curves in Fig 2, which show that conductance changes are small at low voltages (±10 mV), which typically arise during measurements of coupling coefficients. Significant changes of junctional conductance can only be expressed at high voltages (±100 mV), which develop during the spread of excitation. We believe that these observations might have practical applications in electrophysiological experiments when studying the strength and rectification properties of electrical synapses.

The aforementioned sources of functional asymmetry are independent by nature, e.g. R_in_ of a neuron directly depends on plasma membrane area, while synaptic rectification is determined by properties of gap junction channels [48]. Thus, they can act antagonistically promoting bidirectionality of electrical synapses, as was demonstrated in the teleost auditory system [4]. Alternatively, if rectification of gap junctions and differences in R_in_s acted synergistically, it could facilitate unidirectional AP transfer. Thus, unidirectionality, which is a genuine property of chemical synapses, could be executed through electrical synapses alone. Because electrical synaptic transmission is faster than chemical, unidirectional spread of AP through gap junctions might be useful in rapid response warranting behavior such as escape reflex [49, 50].

Asymmetry of electrical synaptic transmission plays an important role in spike-timing regulation, as was demonstrated in neurons of the thalamic reticular nucleus [44]. In larger networks, even a small asymmetry would add up during the spread of excitation and could significantly affect the latency of AP transfer along neural pathways. This process could be crucial in temporal coding activities, such as coincidence detection, in which gap junctions are reported to play an important role [50]. Presumably, the effect of asymmetry of electrical signaling would be difficult to measure and observe experimentally in highly complex neuronal networks, and a simulation-based approach could provide valuable insights on the role of rectification in network dynamics [51].

### The plasticity of electrical synapses and its functional role in shaping the behavior of a neuronal network

It is well established that gap junctional conductance depends on voltage [10]. Our previous [20] and current modeling studies show that decay of junctional conductance can be induced by voltage gating of gap junction channels during bursting activity of neurons. To our knowledge, at least one study reported such spiking activity-dependent reduction of electrical synaptic strength in brain slices [52]. Our data showed that even in gap junctions formed of low-voltage-sensitive Cx36, this decay exceeds 10% while in more voltage-sensitive Cx isoforms it could reach ~50% over several seconds (Figs 6 and 7). The magnitude of junctional conductance decrease and duration of its recovery depends not only on Cx properties but also on the firing rates of neurons (Fig 6). Because the transfer of electrical signal and its asymmetry depends on junctional conductance [53], an activity-induced inhibition of electrical synapses can significantly diminish (Fig 6A-b) or even abolish AP transfer between neurons (Fig 8C-c). Such a role of electrical synaptic plasticity was acknowledged in [54] and was demonstrated by an activity-dependent decrease of junctional conductance together with enhanced asymmetry of electrical synaptic transmission in TRN slices [52].

Heterotypic gap junctions exhibit structure-determined voltage-gating asymmetry, which could result in even more diverse functional behavior with respect to plasticity and directionality than homotypic gap junction. As we showed in Fig 8, changes in junctional conductance and the response rate of neurons depends on the direction of AP spread with respect to the orientation of heterotypic gap junctions. Thus, heterotypic synapses could promote direction-dependent asymmetry of electrical signal transfer not only by its rectification properties but by asymmetric voltage gating as well. We presume that such processes might have an important functional role in sensory systems where heterotypic electrical synapses are detected [55, 56].

Regulation of the strength of electrical synapses by a variety of chemical reagents is well established. Others and our data showed that junctional conductance decay caused by chemical uncouplers can be reversed by voltage, while some chemical factors can change voltage sensitivity of Cxs [8, 38, 39, 57]. These observations, as well as the fact that all known chemical uncouplers close gap junction channels fully but not to residual conductance, suggest that some chemical factors act through the *slow* gate. We implemented this idea by simulating Mg^2+^-mediated changes in junctional conductance of Cx36 gap junctions using the 36SM. The obtained data revealed that an effect of [Mg^2+^]_i_ can be relatively well reproduced (Fig 9) assuming variation in values of 36SM parameters, mainly V_0_ and probabilities of c_1_↔c_2_ transitions of slow gates. Moreover, because the voltage sensitivity of the gap junction channels is defined by the same parameters (see Fig 4 in [20] and Fig 6 in this paper), chemically modulated gating would also affect spiking activity-dependent short-term plasticity of the electrical synapse. Our modeling results showed that even a moderate change (±20%) in [Mg^2+^]_i_ could result in very significant differences in the spread of APs between two neurons (see Fig 10). Thus, chemically modulated gating of Cx36 can expand the time window of electrical synaptic plasticity for as long as chemical factors are present, which could last for minutes or even hours. Therefore, even Cx36, which exhibits relatively low voltage sensitivity, could act as a highly modulatable constituent of neuronal networks due to chemically mediated gating.

Our modeling results show that a persistent spiking activity or chemical factors could keep a significant proportion of gap junction channels in a closed state. We assume that this process could offer at least a partial explanation to a well-documented ‘low functionality’ of gap junctions, especially those expressed in excitable cells, such as neurons or cardiomyocytes. Low functionality refers to a small fraction of channels residing in the open (or high conductance) state. This applies to all connexins, such as Cx36 [58], Cx43 [26], Cx45 [57] and Cx57 [59], examined on this issue, and likely applies to other Cx isoforms.

The strength of electrical synapses directly affects the level of synchronization in neuronal networks, which can underlie various physiological processes and pathological brain conditions. For example, increased cortical synchronization correlates with reduced information processing capability in the primary auditory cortex [60]. The rise in junctional conductance can lead to over-synchronization, which is associated with episodes of epileptic seizures. Interestingly, an activity-induced decrease in the coupling of electrical synapses through an intracellular Ca^2+^ mechanism was observed in the thalamic reticular nucleus of epileptic rats and was proposed to act as a compensatory mechanism to reduce excessive synchronization [61]. Thus, both voltage- and chemically induced gating of gap junction channels can play an important role in shaping activity of neuronal networks through modulation of neuronal synchrony. In addition, short-term plasticity induced through voltage gating of electrical synapses could contribute to lateral inhibition and resulting center-surround effect, which is important in sensory systems of the CNS. This hypothesis is supported by studies showing that more voltage-sensitive Cx isoforms are expressed in the structures associated with sensory functions. For example, one of the most voltage-sensitive Cxs, mouse Cx57 and its human homolog Cx62 are expressed in horizontal cells of the retina [62], while Cx45, which is significantly more voltage-sensitive than Cx36, predominates in the olfactory bulb [34].

The chemically mediated gating could play an important role in regulating longer term changes, especially in less-voltage-sensitive Cx36. For example, it was reported that Cx36 plays an important role in shifting between sleep and wake states [63]. We believe that the unique sensitivity of Cx36 to Mg^2+^ could contribute to this process. This view is supported by accompanying changes in ATP levels, which effectively influence [Mg^2+^]_i_. It was reported that ATP levels increase during the initial hours of sleep in wake-active regions of rat brain [64]. This should decrease [Mg^2+^]_i_ and, consequently, increase conductance of Cx36 gap junctions. As a result, an increased synchronization could suppress activities in brain regions associated with the waking state, thus maintaining sleep.

### Applicability of the proposed modeling approach to other tissues

In this study, we used the Hodgkin–Huxley equations to describe excitability of neurons. The developed model can be adapted to various brain regions and circuits by choosing an appropriate set of ionic currents. For example, the inclusion of Ca^2+^ currents, which underlie bursting trains of APs in thalamic relay neurons [65], might be relevant for short-term plasticity as well as for chemical modulation of electrical synapses. Furthermore, major principles used to develop an HH-36SM can be applied in cardiac tissue modeling, provided that the Hodgkin–Huxley equations are replaced by those specific for cardiomyocytes [66, 67]. Cardiomyocytes are predominantly connected through Cx43, Cx40 and Cx45, which are more voltage sensitive than Cx36; therefore, it might exhibit more expressed activity-dependent conductance decrease, especially during tachyarrhythmias. Furthermore, chemically mediated gating of cardiac gap junction channels, e.g. by acidification [68], could be important in describing enhanced arrhythmogenicity of the ischemic myocardium [69].

### Limitations of the model and further improvements

Obviously, the 36SM of gap junction channel voltage gating is a simplification of complex processes underlying changes of electrical synaptic strength. However, we believe that rectification and voltage gating properties of gap junction channel can be reasonably well reproduced using the 36SM. On the other hand, an inclusion of chemical modulation into 36SM is far less explored. So far we made only the first steps in this direction to explain Cx36 mediation by [Mg^2+^]_i_, and presented modeling results (Fig 10) are obtained from just a few data points. Moreover, cytosolic conditions are rarely defined by a single chemical factor, and various different reagents might affect electrical synapses synergistically or antagonistically. For example, our preliminary data suggest that [Mg^2+^]_i_ effect on Cx36 gap junctions might depend on the pH level. In addition, modulation of electrical synapses by other chemical reagents, such as Ca^2+^ ions, might be more relevant for the spread of excitation than that of [Mg^2+^]_i_.

In this study, we simulated electrical synaptic transmission between two cells connected through a soma-somatic gap junction. For a more realistic neuronal network simulation, it would be beneficial to include dendro-dendritic connections, which are far more prevalent in mammalian brain. Another important extension of our model would be an inclusion of chemical synapses. Presumably, this would allow one to study an interaction between chemical and electrical synapses, which was observed in numerous experimental studies [70].

However, all physiologically relevant extensions, and especially an increased number of cells and synapses, might require a large amount of computational recourses. To our knowledge, Hodgkin-Huxley type models are rarely applied for large neuronal network simulation due to computation time constraints. This problem would be enhanced by our modeling approach, because evaluation of junctional conductance using the 36SM consumes ~95 percent of overall computation time. We presume that simulation time could be decreased by two different approaches: 1) Creation of a more simplistic model of gap junction voltage gating, which would roughly describe relative changes of junctional conductance in response to a single AP. Somewhat similar approach is applied in mathematical models of chemical synapses [71]. This would allow one to combine a model of electrical synapse with integrate-and-fire type models, which are often used for simulation of large neuronal networks. 2) Application of advanced computation techniques, such as an extensive parallelization together with graphic processing unit computation.

## Supporting information

S1 Text Membrane excitability model. (PDF)

S2 Text Fitting 36SM to reproduce an effect of [Mg^2+^]_i_ on junctional conductance of Cx36 gap junction. (PDF)

S1 Fig The decrease of junctional conductance (g_j_) in electrical synapses, induced by neuronal spiking activity. APs in the presynaptic cell were invoked by a series of stimuli, distributed according to a Poisson distribution. The developed transjunctional voltage spikes caused gating of gap junction channels. The accumulated decrease in g_j_ depends on firing rate of coupled neurons, as well as on voltage sensitivity of the gap junction. Here, voltage sensitivity was simulated by changing deep-closed transition probability p_c1→c2_; in (AB-a), p_c1→c2_ was equal to 0.001, while in (AB-b), p_c2→c1_ = 0.0001. (A) Poissonian stimuli of 55 Hz caused ~7.5% g_j_ decay in the less-voltage-sensitive synapse (A-a), while it reached ~25% decrease in a more-voltage-sensitive synapse (A-b) in less than 10 s. (B) The decrease in synaptic strength caused by low firing rate was insignificant because g_j_ had enough time to recover between spikes. Overall, the g_j_ decrease reached only ~1% in the less-voltage-sensitive electrical synapse (B-a), and was lower than 3% in the more-voltage-sensitive synapse (B-b).
(TIF)

S2 Fig Evaluation of Cx36 voltage-gating parameters values at different levels of [Mg^2+^]_i_. The approximated values of 36SM parameters V_0_ (A), p_c1→c2_ (B) and ratio p_c1→c2_/p_c2→c1_ (C) at different levels of [Mg^2+^]_i_. Black circles denote parameter values, which were obtained from electrophysiological experiments. White circles denote data points estimated from fitted curves.
(TIF)

S1 Dataset Data for Figs 2–6 and Figs 8 and 9. (XLSX)

S2 Dataset Data for Fig 7A. (XLSX)

S3 Dataset Data for Fig 7B. (XLSX)

S4 Dataset Data for Fig 10 and S1 and S2 Figs. (XLSX)
