# Supplementary material for: Functional asymmetry and plasticity of electrical synapses interconnecting neurons through a 36-state model of gap junction channel gating
Source: PLoS Comput Biol. 2017 Apr 6;13(4):e1005464. doi: 10.1371/journal.pcbi.1005464 (PMC5398722; doi:10.1371/journal.pcbi.1005464)
Supplement: S1 Text — (PDF) [file pcbi.1005464.s001.pdf]

Membrane excitability model

We have used an original form of the Hodgkin–Huxley model, just with added junctional current and shifted resting potentials. It can be described by the following system of ordinary differential equations:

$$\begin{cases} C_m \dfrac{dV_m}{dt} = A \cdot \left(g_K \cdot n^4 \cdot (V_m - V_K) + g_{Na} \cdot m^3 \cdot h \cdot (V_m - V_{Na}) + g_l \cdot (V_m - V_l)\right) + I_j - I_{ext}; \\ \dfrac{dn}{dt} = \alpha_n(V_m) \cdot (1-n) - \beta_n(V_m) \cdot n; \\ \dfrac{dm}{dt} = \alpha_m(V_m) \cdot (1-m) - \beta_m(V_m) \cdot m; \\ \dfrac{dh}{dt} = \alpha_h(V_m) \cdot (1-h) - \beta_h(V_m) \cdot h. \end{cases}$$

Here C_m is the membrane capacitance; V_m is membrane potential; t is the time; g_K, g_{Na} and g_l are the maximum values of potassium, sodium and leak conductances per unit area, respectively; n, m and h are variables ($0 \le n, m, h \le 1$) associated with potassium channel activation, sodium channel activation and sodium channel inactivation, respectively; V_K, V_{Na} and V_l are the potassium, sodium and leak reversal potentials, respectively; I_i is transjunctional current; I_{ext} denotes applied external current; α_i and β_i are rate constants for the respective ion channel evaluated at given V_m.

The membrane capacity C_m was estimated from $C_m = C{\cdot}A$, where C is the specific membrane capacitance and is equal to 1 μF/cm^2, while A denotes the surface area of the plasma membrane. For simplicity, we assumed that the cell membrane has the same surface area as a sphere with radius r, which in most cases was equal to 10 μm.

The maximum conductances of ion channels were chosen as in the original model: g_K = 36 mS/cm^2, g_{Na} = 120 mS/cm^2 and g_l = 0.3 mS/cm^2.

The resting membrane potentials were shifted to –70 mV, as compared with the original model, therefore reversal potentials were as follows: V_K = –82, V_{Na} = 45, V_l = –59.4 mV. The rate constants α_i and β_i were adjusted accordingly:

$$\alpha_n(V_m) = \frac{0.8 - 0.01 \cdot V_m}{e^{(8 - 0.1 \cdot V_m)} - 1}; \ \alpha_m(V_m) = \frac{9.5 - 0.1 \cdot V_m}{e^{(9.5 - 0.1 \cdot V_m)} - 1}; \ \alpha_h(V_m) = 0.07 \cdot e^{(-0.05 V_m - 3.5)};$$

$$\beta_n(V_m) = 0.125 \cdot e^{(-0.0125 V_m - 0.875)}; \ \beta_m(V_m) = 4 \cdot e^{\left(\frac{-V_m - 70}{18}\right)}; \ \beta_h(V_m) = \frac{1}{e^{(10 - 0.1 \cdot V_m)} + 1}.$$

Junctional current I_j between two cells (cell-1 and cell-2) was estimated as $I_j = g_j(V_j) \cdot (V_2 - V_1)$ for cell-1, and as $I_j = g_j(V_j) \cdot (V_1 - V_2)$ for cell-2. Junctional conductance $g_j(V_j)$ at a given transjunctional voltage, $V_j = (V_2 - V_1)$, was evaluated from the 36-state model (36SM).

We used Euler's method for the numerical solution of the Hodgkin–Huxley equations, which was implemented in MATLAB.
