# Supplementary material for: Functional asymmetry and plasticity of electrical synapses interconnecting neurons through a 36-state model of gap junction channel gating
Source: PLoS Comput Biol. 2017 Apr 6;13(4):e1005464. doi: 10.1371/journal.pcbi.1005464 (PMC5398722; doi:10.1371/journal.pcbi.1005464)
Supplement: S2 Text — (PDF) [file pcbi.1005464.s002.pdf]

Fitting 36SM to reproduce an effect of intracellular free magnesium ion concentration ($[Mg^{2+}]_i$) on junctional conductance (g_j) of Cx36 gap junction

Model fitting was performed, assuming that variation in $[Mg^{2+}]_i$ changes the values of the 36SM parameters. We used the following values of 36SM parameters at basic level of $[Mg^{2+}]_i$ equal to 1 mM: A = 0.15 mV^{-1}, V_0 = 19 mV, $p_{c1\to c2}$ = 0.015 and $p_{c2\to c1}$ = 0.001. Because we assumed non-rectifying gap junction channels, rectification coefficients $R_{F,open}$, $R_{F,res}$ and $R_{S,open}$ were set at 10^6 mV. The kinetics of 36SM parameter changes under $[Mg^{2+}]_i$ = 0.01 and 5 mM were modelled by a logistic function of the following form:

$$P(t) = \frac{P_{\max} \cdot P_0}{P_0 + (P_{\max} - P_0) \cdot e^{-\alpha \cdot t}} . \quad (1)$$

Here, $P(t)$ denotes the value of 36SM parameter at time moment t, P_{max} is a steady-state value of the 36SM model parameter reached at a given level of $[Mg^{2+}]_i$, P_0 denotes initial values of the 36SM parameters at $[Mg^{2+}]_i$ = 1 mM, α is the steepness of the logistic curve.

The optimized values of 36SM and logistic curve parameters fitted to junctional conductance changes under $[Mg^{2+}]_i$ = 0.01 and 5 mM are presented in Table S1.

Table S1: Values of model parameters obtained from global optimization during fitting junctional conductance changes under different $[Mg^{2+}]_i$.

$[Mg^{2+}]_i$, mM	A, mV^{-1}	V_0, mV	$p_{c1\to c2}$	$p_{c2\to c1}$	α, s^{-1}
0.01	0.15010	92.5	0.000009	0.00156	0.00089
5	0.15006	7.21	0.958	0.0334	0.055

To evaluate junctional conductance at more physiological levels, we approximated possible values of V_0, $p_{c1\to c2}$ and $p_{c2\to c1}$ from optimized values at $[Mg^{2+}]_i$ = 0.01, 1 and 5 mM. The parameter A was fixed at 0.15 mV^{-1} because its changes were insignificant. The transition probability $p_{c2\to c1}$ was estimated from approximated values of $p_{c1\to c2}$ and the ratio $p_{c1\to c2}/p_{c2\to c1}$. To estimate V_0 values at different $[Mg^{2+}]_i$ we used an exponential decay curve (see also S2 Fig):

$$V_0([Mg^{2+}]_i) = y_0 + a \cdot e^{-b \cdot [Mg^{2+}]_i} . \quad (2)$$

The values of y_0, a and b were estimated using Sigmaplot curve fitting tools. Here, y_0 = 7.2 mV, a = 87 mV and b = 2 mM^{-1}.

Transition probabilities $p_{c1\to c2}$ were approximated (see also S2 Fig) by a logistic function as follows:

$$p_{c1\to c2}([Mg^{2+}]_i) = \frac{a}{1 + \left(\frac{[Mg^{2+}]_i}{x_0} \right)^b} . \quad (3)$$

Here, a = 1.13, b = –3.74 and x_0 = 3.16 mM.

Finally, the ratio $p_{c1\to c2}/p_{c2\to c1}$ was approximated by an exponential rise to a maximum level (see also S2 Fig):

$$\frac{p_{c1\to c2}}{p_{c2\to c1}}([Mg^{2+}]_i) = a \cdot \left(1 - e^{-b \cdot [Mg^{2+}]_i}\right), \quad (4)$$

with a = 29.54 and b = 0.71 mM^{-1}.

The approximated values of steady-state 36SM parameters at $[Mg^{2+}]_i$ = 0.8 and 1.2 mM are presented in Table S2.

Table S2. Approximated steady-state values of 36SM parameters under free intracellular magnesium ion concentration ($[Mg^{2+}]_i$) of 0.8 and 1.2 mM.

$[Mg^{2+}]_i$, mM	V_0, mV	$p_{c1 \rightarrow c2}$	$p_{c2 \rightarrow c1}$
0.8	24.7953	0.00656	0.00051
1.2	15.1141	0.02929	0.00173
