# Supplementary figures and images for: Functional asymmetry and plasticity of electrical synapses interconnecting neurons through a 36-state model of gap junction channel gating
Source: PLoS Comput Biol. 2017 Apr 6;13(4):e1005464. doi: 10.1371/journal.pcbi.1005464 (PMC5398722; doi:10.1371/journal.pcbi.1005464)

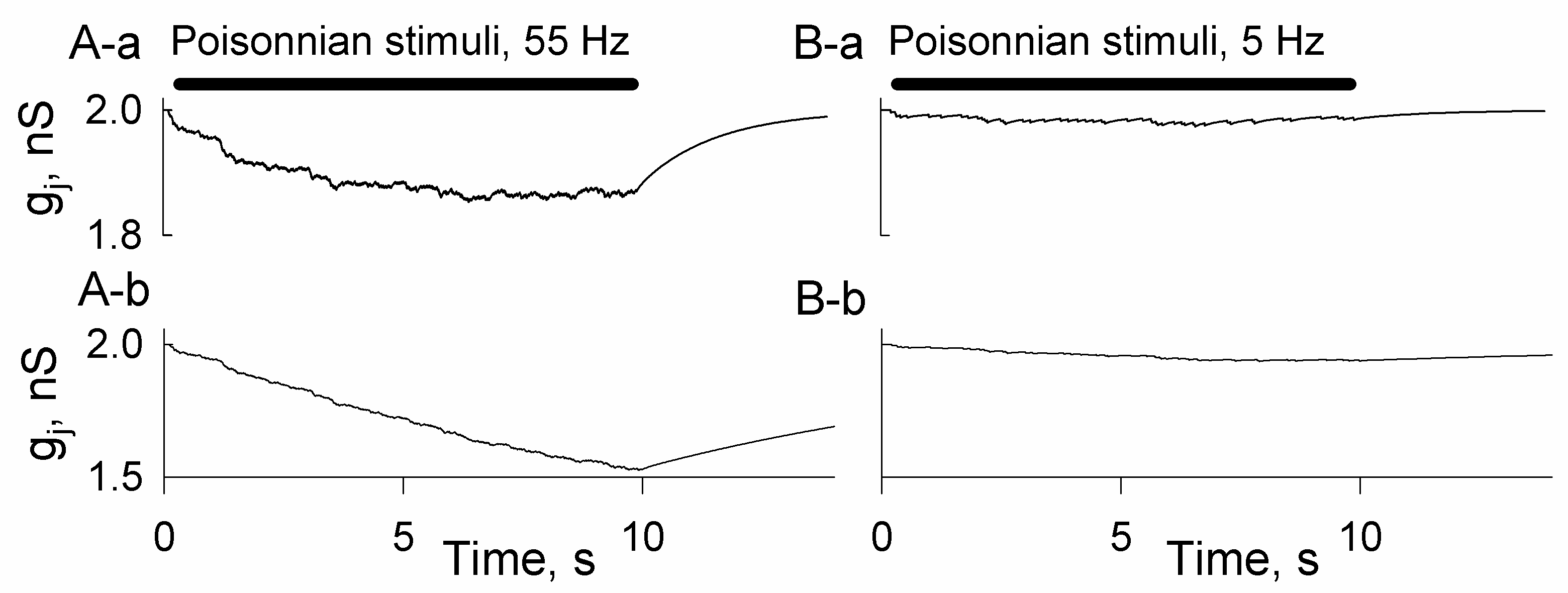

Supplement: S1 Fig — APs in the presynaptic cell were invoked by a series of stimuli, distributed according to a Poisson distribution. The developed transjunctional voltage spikes caused gating of gap junction channels. The accumulated decrease in gj depends on firing rate of coupled neurons, as well as on voltage sensitivity of the gap junction. Here, voltage sensitivity was simulated by changing deep-closed transition probability pc1→c2; in (AB-a), pc1→c2 was equal to 0.001, while in (AB-b), pc2→c1 = 0.0001. (A) Poissonian stimuli of 55 Hz caused ~7.5% gj decay in the less-voltage-sensitive synapse (A-a), while it reached ~25% decrease in a more-voltage-sensitive synapse (A-b) in less than 10 s. (B) The decrease in synaptic strength caused by low firing rate was insignificant because gj had enough time to recover between spikes. Overall, the gj decrease reached only ~1% in the less-voltage-sensitive electrical synapse (B-a), and was lower than 3% in the more-voltage-sensitive synapse (B-b). (TIF) [file pcbi.1005464.s003.tif]

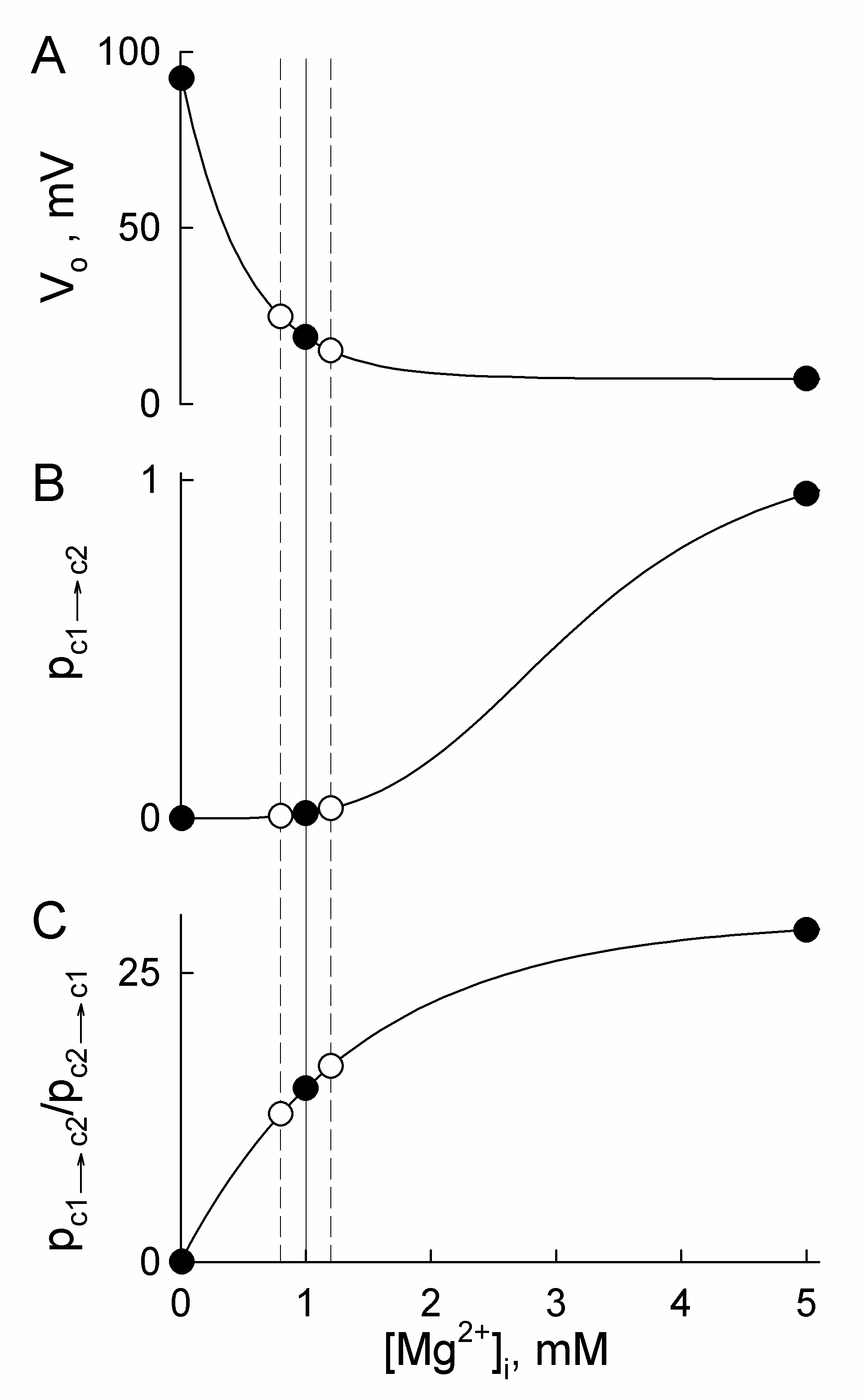

Supplement: S2 Fig — The approximated values of 36SM parameters V0 (A), pc1→c2 (B) and ratio pc1→c2/pc2→c1 (C) at different levels of [Mg2+]i. Black circles denote parameter values, which were obtained from electrophysiological experiments. White circles denote data points estimated from fitted curves. (TIF) [file pcbi.1005464.s004.tif]
